# Selective attention in rat visual category learning

**DOI:** 10.1101/lm.048942.118

**Published:** 2019-03

**Authors:** Matthew B. Broschard, Jangjin Kim, Bradley C. Love, Edward A. Wasserman, John H. Freeman

**Affiliations:** 1 Department of Psychological and Brain Sciences, University of Iowa, Iowa City, Iowa, 52242, USA; 2 Department of Experimental Psychology and The Alan Turing Institute, University College London, London WC1H 0AP, United Kingdom

## Abstract

A prominent theory of category learning, COVIS, posits that new categories are learned with either a declarative or procedural system, depending on the task. The declarative system uses the prefrontal cortex (PFC) to learn rule-based (RB) category tasks in which there is one relevant sensory dimension that can be used to establish a rule for solving the task, whereas the procedural system uses corticostriatal circuits for information integration (II) tasks in which there are multiple relevant dimensions, precluding use of explicit rules. Previous studies have found faster learning of RB versus II tasks in humans and monkeys but not in pigeons. The absence of a learning rate difference in pigeons has been attributed to their lacking a PFC. A major gap in this comparative analysis, however, is the lack of data from a nonprimate mammalian species, such as rats, that have a PFC but a less differentiated PFC than primates. Here, we investigated RB and II category learning in rats. Similar to pigeons, RB and II tasks were learned at the same rate. After reaching a learning criterion, wider distributions of stimuli were presented to examine generalization. A second experiment found equivalent RB and II learning with wider category distributions. Computational modeling revealed that rats extract and selectively attend to category-relevant information but do not consistently use rules to solve the RB task. These findings suggest rats are on a continuum of PFC function between birds and primates, with selective attention but limited ability to utilize rules relative to primates.

A model of human category learning, COVIS (competition between verbal and implicit systems), posits that new categories are learned by two functionally and anatomically distinct neural systems: a declarative system and a procedural system (Ashby et al. 1998; Ashby and Maddox 2005, 2010). Assumed to be phylogenetically older, the procedural system uses associative learning mechanisms to map category stimuli to appropriate behavioral responses. Learning here is an incremental process and relies on reinforcement. This system is robust and capable of learning most category structures, as long as immediate feedback is available. The declarative system uses executive functions (e.g., working memory and selective attention) to develop and utilize rules of the category task. Under this system, the learner explicitly tests hypotheses about potential rules, and feedback provides information regarding the validity of each rule. Compared to the procedural system, the declarative system is more limited in the tasks that it can learn. Suitable structures for the declarative system entail category stimuli containing irrelevant information, where the same dimensional value appears in exemplars of multiple categories. For these structures, a successful category rule can be generated by using selective attention to category-relevant information (in which dimensional values are exclusive to one category) and ignoring category-irrelevant information (in which dimensional values occur in more than one category).

To observe behavioral and neural dissociations between the declarative and procedural systems, a line of research has compared rule-based (RB) and information integration (II) learning tasks (Fig. 1; Maddox and Ashby 2004; Nomura et al. 2007; Maddox et al. 2009; Smith et al. 2013, 2015a,b; Soto et al. 2013). Exemplars are discs containing black and white gratings that change in their spatial frequency and orientation. A category of discs is created by placing a normal distribution on this two-dimensional space (frequency and orientation). On each trial, participants are presented with an exemplar generated from a distribution and must decide its category membership. Typically, feedback is provided after each trial and training continues until a learning criterion is met or after a set number of trials.

**Figure 1.**
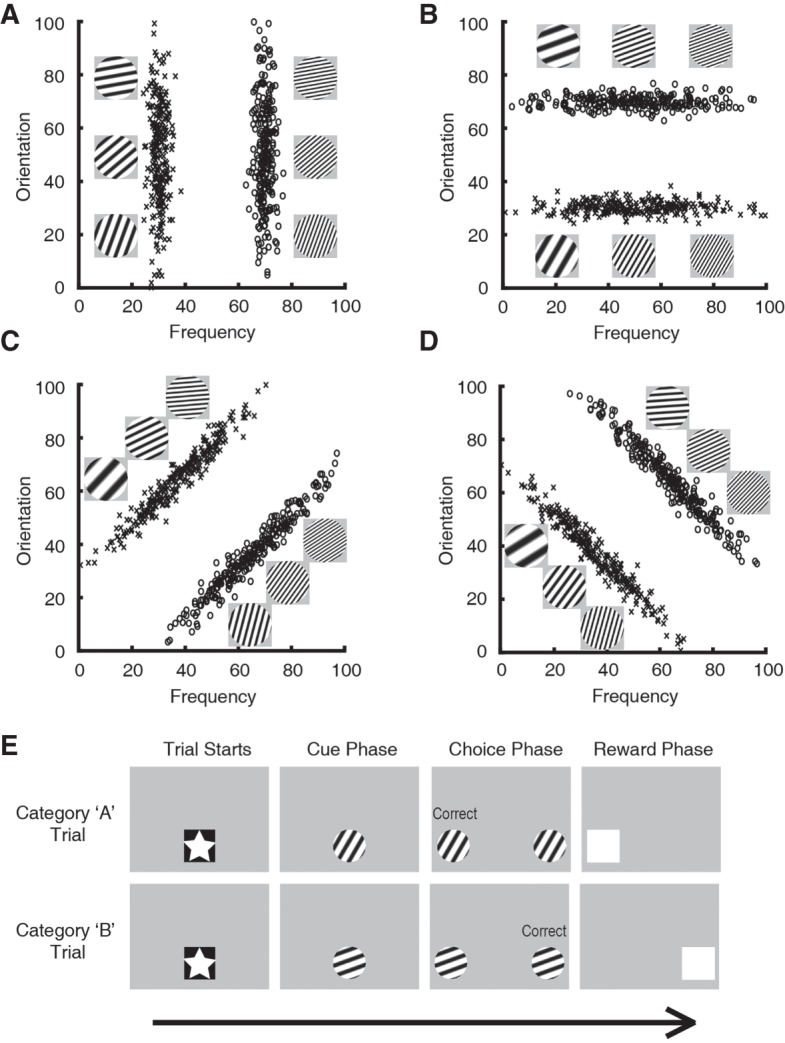
(*A–D*) Category tasks used in Experiment 1. Each point represents a category exemplar with line gratings of particular spatial frequency and orientation. Each distribution constitutes a category. (*A*,*B*) Rule-based (RB) category tasks can be solved by creating a one-dimensional rule. (*C*,*D*) Information integration (II) tasks are 45° rotations of the RB tasks. Here, a unidimensional rule cannot segregate the categories. (*E*) Trial sequence during training and testing sessions. Rats initiated each trial by touching the star cue (star phase). Then, the rat touched the grating stimulus three times (cue phase), at which point this same stimulus was presented on the left and right sides of the screen acting as response keys (choice phase). Members of category A required a touch to the left response key, whereas members of category B required a touch to the right response key. A correct choice produced a white box and food reward. An incorrect choice initiated a correction trial.

RB tasks entail category distributions that are perpendicular to one of the axes (in Fig. 1A, spatial frequency; in Fig. 1B, orientation), whereas the II tasks entail 45° rotations of the RB distributions (Fig. 1C,D). This rotation does not change any intrinsic property of the task (e.g., the RB and II tasks are identical in their logical separability and difficulty); however, the II tasks are no longer perpendicular to an axis. This difference is key. The RB tasks can be solved by generating a unidimensional rule (i.e., attending to the dimension that is perpendicular to the distributions), but the optimal boundary for II tasks is a diagonal line; thus, solving II tasks requires combining information from both dimensions. RB tasks can be learned using the declarative system, but II tasks must be learned by the procedural system.

Humans can learn RB tasks faster than II tasks, which is possible if the learner uses selective attention to find the unidimensional category rule in the RB task (Smith et al. 2012). In a comparative analysis, macaque and capuchin monkeys also learned RB tasks faster than II tasks, but pigeons learned both tasks at the same rate (Smith et al. 2012). The interpretation was that primates, but not avians, utilize a second learning system, the declarative system, to find category rules. This species difference may importantly inform the evolutionary trajectory of category learning (Smith et al. 2012), with primates able to represent RB tasks using rules, but avians forced to deploy a single associative learning system.

An alternative interpretation of the species difference is that all species have a single learning system that adjusts selective attention based on the demands of the task (Nosofsky 1986; Kruschke 1992; Rehder and Hoffman 2005). Accordingly, RB tasks require selective attention to the relevant dimension, whereas II tasks can be solved with attention divided between dimensions. The lack of an RB learning advantage in avians might therefore be related to weaker selective attention relative to primates, rather than to the absence of the declarative system.

A major gap in our understanding of category learning is whether the RB advantage is exclusive to primates. Specifically, would nonprimate mammals, such as rats, learn the RB tasks faster than the II tasks? On the one hand, one might predict that rats would show a learning advantage for the RB tasks, as classic behavioral paradigms have demonstrated that rat cognition supports the executive functions described by COVIS's declarative system, including working memory and selective attention (Bruin et al. 1994; Horst and Laubach 2009). Furthermore, rats have learned visual categories that require attention to single relevant features among irrelevant information, suggesting that selective attention can be used in category learning (Wasserman et al. 2012; Brooks et al. 2013; Vinken et al. 2014; Kim et al. 2018).

On the other hand, the extent to which the executive functions of rats are comparable to those of primates is not clear. This issue lies in the functional contributions to cognition of the prefrontal cortex (PFC), the central brain region of the declarative system in the COVIS model. COVIS posits that the PFC identifies, selects, and applies each category rule (Ashby et al. 1998). Converging evidence supports the role of the PFC in rule use and attributes this function to the lateral PFC (lPFC) in primates (Wutz et al. 2018). Although the rodent PFC mediates similar cognitive functions as the primate PFC, there are key anatomical differences in the cellular makeup and organization between these orders of animals (Uylings and Eden 1991; Seamans et al. 2008; van Aerde and Feldmeyer 2013). Furthermore, the anatomy of the rodent PFC is more similar to the primate's medial PFC (mPFC). By ascertaining whether rats learn RB tasks faster than II tasks, we may gain considerable insight into the functional similarity of the rodent PFC and the primate PFC; specifically, we can suggest when rule use emerged phylogenetically.

In the current study, we trained rats to learn RB and II tasks in two experiments in our search for task differences. In Experiment 1, male and female rats were trained on either an RB task or an II task. Upon reaching a learning criterion, rats were given generalization testing where stimuli were generated from broader distributions containing novel exemplars. Decision boundary models were tested on the generalization data to determine the rats’ strategies during both tasks (Maddox and Ashby 1993). Additionally, the generalized context model (GCM) was fit to the generalization data to examine the allocation of attention during RB and II tasks (Nosofsky 1986). Importantly, this analysis sought to determine whether the rats use selective attention when learning the RB tasks. In Experiment 2, we trained male rats to learn RB and II tasks with more variable distributions in order to increase the load on their selective attention system. Decision boundary models were also fit to the data in Experiment 2.

## Results

### Experiment 1

#### Training

**Figure 2.**
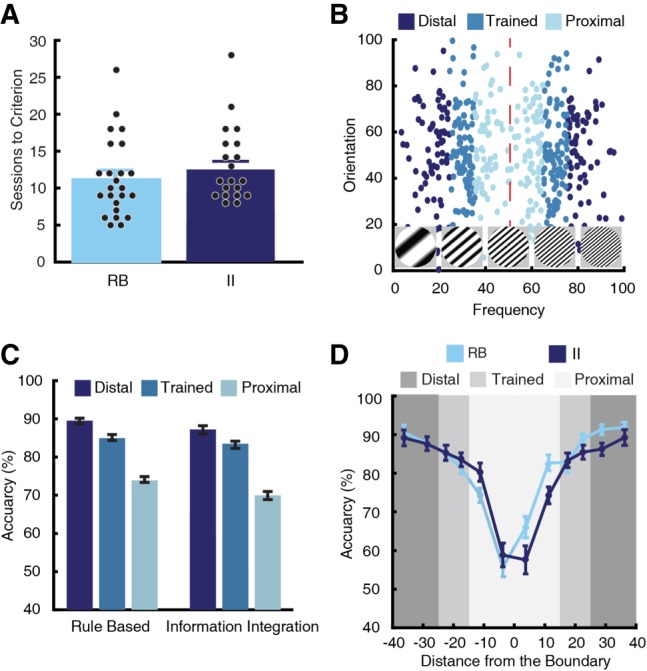
(*A*) The mean number of sessions (±SEM) for rats to reach the learning criterion. There was no difference in sessions to criterion between rats learning the RB and II tasks. (*B*) Testing distributions for a RB task used in Experiment 1. Compared with the training distributions, testing distributions had identical means but larger SD. Thus, the testing distributions were segregated into three trial types: stimuli that were within two SDs of the training distributions (Trained), stimuli beyond two SDs of the training distributions and closer to the category boundary (Proximal), and stimuli beyond two SDs of the training distributions and farther from the category boundary (Distal). (*C*) Performance on the testing distributions according to trial type. For both RB and II tasks, accuracy increased for Distal stimuli and decreased for Proximal stimuli compared to Trained stimuli. (*D*) Accuracy was calculated according to distance from the category boundary. For both RB and II tasks, performance increased as a function of distance from the category boundary.

In the current experiment, rats were trained to learn either an RB or an II task in search of task differences. All but one female rat (learning an II task) reached the acquisition criterion. This rat did not reach accuracies above chance after 40 sessions. The remaining rats were included in all analyses. Using a 2 × 2 between-subjects ANOVA, we examined the effects of sex and task type on the number of sessions to reach criterion. There was a main effect of sex, such that females took significantly more sessions to reach criterion than males (Supplemental Fig. S1A; *F*_(1,43)_ = 8.25, *P* = 0.006). Importantly, there was no significant main effect of task type or sex by task type interaction (*F*_(1,43)_ = 0.79, *P* = 0.379; *F*_(1,43)_ = 0.078, *P* = 0.782, respectively). Thus, the RB tasks and II tasks were learned at the same rate (Fig. 2A).

To examine accuracy across training, sessions were vincintized into five training blocks, so that each rat contributed an equal number of samples to the analysis (see Materials and Methods). Then, a linear mixed-effects model compared accuracy across training blocks. The model included fixed effects of sex, task type (RB or II), training block, a quadratic function (across blocks), and random effects for the intercept, slope, and the quadratic function. The quadratic function was added to the model because it fit the individual rat learning curves better than the linear function. There was a highly significant main effect for block, such that accuracy improved across training blocks (*t*_(51.48)_ = 8.06, *P* < 0.001). The main effect of task type was not significant (*t*_(44.84)_ = 0.69, *P* = 0.496), so accuracy was not different across training between RB and II tasks. There was a main effect for sex (Supplemental Fig. S1B; *t*_(46.04)_ = 2.22, *P* = 0.032), such that accuracy was lower for females than males across training. However, the interaction between sex and task type as well as the interaction between sex and training block were not significant (*t*_(56.34)_ = 0.06, *P* = 0.950; *t*_(168.89)_ = 0.61, *P* = 0.540, respectively). Thus, females had lower accuracy overall; however, both sexes learned the RB and II tasks at the same rate and to equal levels.

#### Generalization

After reaching the learning criterion, rats completed five sessions categorizing stimuli generated from new distributions. Relative to their training distributions, the testing distributions had identical category means, but greater SDs along the relevant dimension(s) (Fig. 2B). Expanding the SD created three stimulus types: stimuli that overlapped with the training distributions (Trained), novel stimuli that were closer to the category boundary (Proximal), and novel stimuli that were farther away from the category boundary (Distal).

We analyzed category generalization using a linear mixed-effect model. The full linear model included fixed effects for sex, trial type (Distal, Trained, and Proximal), and task type, and random effects for intercept and slope. As in training, we found a significant main effect of sex, such that female rats had lower accuracy during generalization compared to males (Supplemental Fig. S2; *t*_(44.00)_ = 2.42, *P* = 0.020). However, neither the interaction between sex and task type nor the interaction between sex and trial type were significant (*t*_(59.17)_ = 0.18, *P* = 0.856; *t*_(45.17)_ = 1.08, *P* = 0.286, respectively). Therefore, females had lower accuracies during generalization compared to males, but the patterns of generalization were equivalent between the sexes. The main effect of task type as well as the interaction between task type and trial type were not significant (Fig. 2C; *t*_(63.78)_ = 1.33, *P* = 0.189; *t*_(47.39)_ = 1.07, *P* = 0.290). Thus, generalization was equivalent for the RB tasks and II tasks. Finally, there was a significant main effect of trial type (*t*_(48.41)_ = 3.26, *P* = 0.002); pairwise comparisons revealed that performance improved as a function of distance from the decision boundary. Specifically, relative to Trained stimuli, accuracy increased for Distal stimuli and accuracy decreased for Proximal stimuli (Fig. 2C; *P* < 0.001 for both).

This effect of the category boundary also held when the stimuli were more granularly organized according to their distance from the boundary. For this analysis, the stimulus space was rotated so stimuli from all distributions were oriented in the same direction. Stimuli were then binned depending on their distance from the category boundary; we used two bins for each trial type within each category. There was a clear effect of the category boundary, such that accuracy improved as the distance from the center of the categories increased (Fig. 2D). This effect has been observed in human category learning, but until now has not been documented in rats (Davis and Love 2009; Maddox and Filoteo 2011; Seger et al. 2015). Again, the generalization functions were similar for the RB and II tasks.

#### Decision boundary analysis

General recognition theory (GRT) was used to estimate decision boundaries in the generalization data (Fig. 4; Maddox and Ashby 1993). Importantly, this analysis allowed us to infer which strategy each rat was using at testing (i.e., a RB or II strategy; see Materials and Methods for more information). Two RB models that estimate decision boundaries using information from a single stimulus dimension and one II model that uses both stimulus dimensions to estimate a decision boundary were fit to each rat's data. The model of best fit was chosen according to the model with the smallest Akaike's information criterion (AIC) value (Akaike 1974). Roughly half (10/24) of the rats that learned an RB task were best fit to an RB model, with the other half (14/24) best fit by the II model. Therefore, slightly less than half of the rats learning the RB tasks made category decisions according to one stimulus dimension; the other RB rats used both stimulus dimensions to inform their decisions. All (23/23) of the rats that learned the II tasks were best fit to the II model, and therefore had bidimensional decision boundaries.

**Figure 3.**
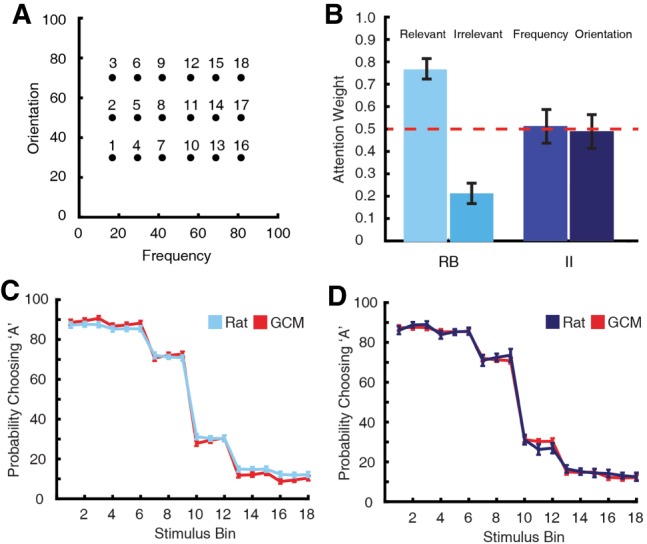
The GCM was fit to the generalization data of Experiment 1. (*A*) First, the testing distributions were averaged into 18 stimulus bins. This included three bins per trial type and nine bins per category. For each bin, the proportion of trials the rat responded category “A” was calculated. (*B*) The estimated attention weights obtained from the GCM fits. RB rats allocated more attention to the relevant stimulus dimension than the irrelevant dimension, whereas II rats split attention evenly between the two stimulus dimensions. (*C*,*D*) Comparison between rat's generalization behavior and GCM's predictions for RB rats (*C*) and II rats (*D*).

#### Generalized context model

The exemplar model GCM was also fit to each rat's generalization data (see Materials and Methods). GCM has been an effective tool in describing human category learning behavior and can assess the degree of selective attention (Mack et al. 2016). To fit GCM, the model was trained with stimuli from each rat's last two sessions of training and then tested with stimuli from the rat's generalization sessions. The model's predictions were fit to the rat's responses by optimizing the model's three free parameters. Overall, the model fit the generalization data well (Fig. 3C,D; mean *R*^2^= 0.889).

Here, the analysis focused on the attention weight parameter, *w*. Each perceptual dimension was given an estimated attention weight (0 < *w* < 1), where all weights add to 1 (*w*_frequency_ + *w*_orientation_ = 1). These weights stretch and shrink dimensions, such that distances along each dimension are exaggerated or attenuated. This scaling mimics selective attention by prioritizing changes along some dimensions over others. For the present experiment, the optimal strategy for learning an RB task would be to allocate all attention to the relevant dimension while ignoring the irrelevant dimension (*w* = 1 and 0, respectively). Conversely, an II task should be learned by equally dividing attention to both dimensions (*w* = 0.5 for both dimensions). Indeed, this is just what the rats did. Using a univariate ANOVA, the mean attention weights were different between the task types (Fig. 3B; *F*_(1,45)_ = 8.64, *P* = 0.005). Using one-sample *t*-tests, the mean attention weights for RB rats were significantly different from 0.5 (*t*_(23)_ = 5.96, *P* < 0.001), whereas the mean attention weights for II rats were not different from 0.5 (*t*_(22)_ = 0.08, *P* = 0.937).

**Figure 4.**
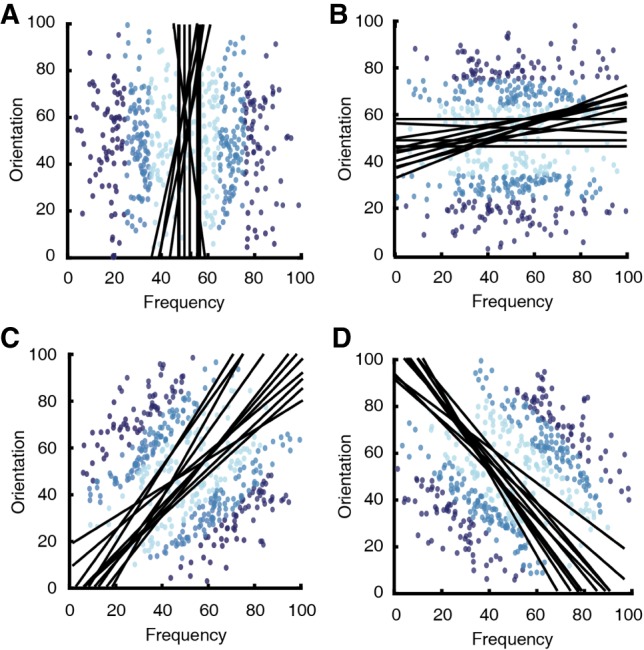
Estimated decision boundaries obtained from GRT. Three models were fit to each rat's generalization data: two models that assume a unidimensional decision boundary (synonymous to an RB strategy) and one that assumes a bidimensional decision boundary (synonymous to an II strategy). The best fit was determined according to the model with the lowest AIC value. (*A*,*B*) The best fitting decision boundaries for rats learning an RB task. (*C*,*D*) The best fitting decision boundaries for rats learning an II task.

The difference in attention weights across tasks is important for two reasons. First, it demonstrates that even though the rats learned the RB and II tasks at the same rate, the tasks were learned differently. Rats used selective attention to the relevant dimension to learn the RB tasks, but they used diffuse attention to learn the II tasks. Second, this finding helps rule out alternative explanations for why the rats learned the tasks at the same rate. Specifically, rats were able to perceive and utilize each dimension separately, so equal learning rates were not a result of perceptual limitations.

### Experiment 2

In Experiment 1, rats learned to categorize RB tasks at the same rate as II tasks. Additionally, category generalization was equivalent between the tasks. Together, these findings indicate that, like pigeons, but unlike primates, rats did not quickly identify and utilize the category rule in the RB tasks. However, the results of the GCM model fittings suggest that the RB rats extracted category-relevant information.

Experiment 2 was conducted to rule out the possibility that no differences were observed in learning rate between RB and II tasks because of a ceiling effect. The rats in Experiment 1 reached the learning criterion very quickly, especially compared to other categorization tasks using the same trial procedures (Brooks et al. 2013; Kim et al. 2018). This may have made it difficult to detect differences in learning between the task types. Therefore, Experiment 2 trained rats to categorize stimuli that covered a larger portion of the stimulus space. Specifically, rats were trained with the same testing distributions that had been used in Experiment 1. This manipulation should make segregating the categories more difficult, because it increases within-category distance and decreases between-category distance (Minda and Smith 2001). This manipulation should strain selective attention, which is especially critical for identifying stimuli near the category boundary.

#### Training

**Figure 5.**
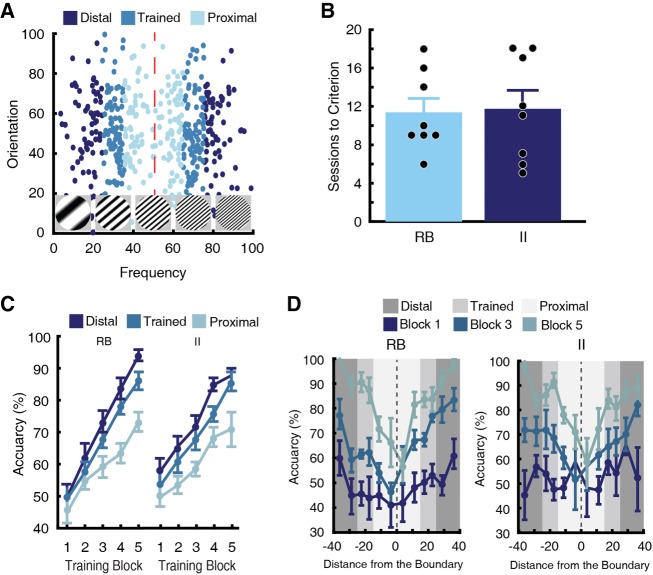
(*A*) Category tasks used in Experiment 2. These distributions had identical means and SDs to the testing distributions of Experiment 1. (*B*) The mean number of sessions to reach the learning criterion for rats learning the RB and II tasks. Similar to Experiment 1, there was no difference in the rate of learning between tasks. (*C*) Accuracy across training for rats learning RB and II tasks. The training sessions were first vincintized so that each rat's learning curve was averaged into five training blocks. There was no difference in accuracy across training between rats learning RB tasks and rats learning II tasks. For both groups, accuracy increased for Distal stimuli and decreased for Proximal stimuli compared to Trained stimuli. (*D*) Accuracy was calculated according to the distance from the category boundary at difference stages of the animal's training (Blocks 1, 3, and 5). For rats learning RB tasks (left) and II tasks (right), performance started at chance. By the end of training, accuracy increased as a function of distance from the category boundary.

Male rats (*n* = 16) were trained to learn the testing distributions of Experiment 1 (Fig. 5A). All rats reached criterion (75% accuracy for both categories on two consecutive sessions) and were included in all analyses. Using an independent *t*-test, the number of sessions to reach criterion did not differ significantly between the task types (Fig. 5B; *t*_(14)_ = 0.75, *P* = 0.466). Training sessions were vincintized into five blocks, and a linear mixed-effects model (fixed effects: task type, training block, a quadratic function; random effects: intercept, slope, and the quadratic function) compared accuracy across training. There was a main effect of training block, such that accuracy increased across training blocks (*t*_(14.00)_ = 3.48, *P* = 0.003). However, there was no significant main effect of task type or interaction between task type and training block (*t*_(14.00)_ = 1.65, *P* = 0.122; *t*_(14.00)_ = .29, *P* = 0.777, respectively). Therefore, no differences were seen in accuracy between task types across training.

Because we used the testing distributions of Experiment 1, we were able to split the distributions into three trial types (Distal, Trained, and Proximal) as before. A linear mixed-effects model (fixed effects: task type, trial type, training block, a quadratic function (across blocks); random effects: intercept, slope, and the quadratic function) compared learning. There was a main effect of training block, such that accuracy increased across training (*t*_(184.49)_ = 4.78, *P* < 0.001). The task type effect as well as the interaction between task type and training block were not significant (*t*_(42.19)_ = 1.97, *P* = 0.078; *t*_(182.61)_ = 1.13, *P* = 0.260). There was a significant effect of trial type (*t*_(112.53)_ = 2.186, *P* = 0.031), such that accuracy for Distal stimuli was higher than accuracy for Trained and Proximal stimuli, and accuracy for Proximal stimuli was lower than accuracy for Trained and Distal stimuli (Fig. 5C; *P* < 0.05 for each).

As in Experiment 1, accuracy was binned according to distance from the category boundary (Fig. 5D). This analysis was repeated at difference stages of training. Notice that accuracy was relatively flat at the beginning of training but quickly developed into the characteristic “V” shape, where accuracy improved with distance from the category boundary. This was true for both RB and II tasks.

#### Decision boundary analysis

**Figure 6.**
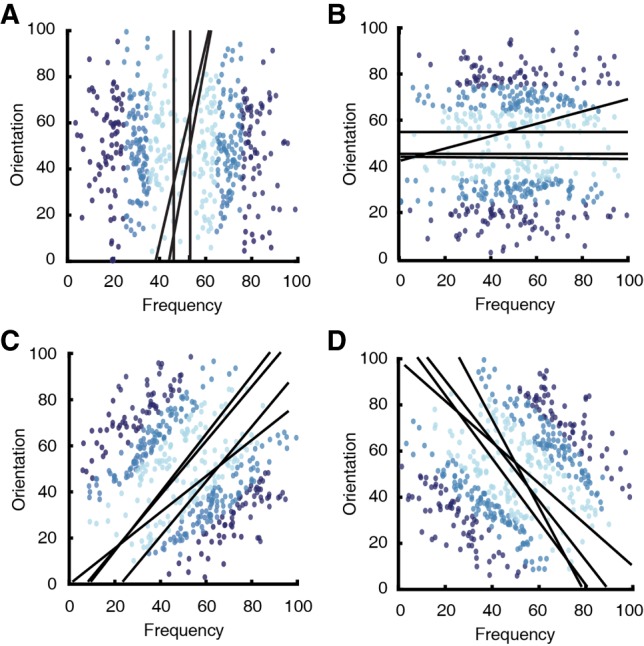
Estimated decision boundaries obtained from GRT. Three models were fit to each rat's generalization data: two models that assume a one-dimensional decision boundary (synonymous to a RB strategy) and one model that assumes a two-dimensional decision boundary (synonymous to an II strategy). The best fit was determined according to the model with the lowest AIC value. (*A*,*B*) The best fitting decision boundaries for rats learning a RB task. (*C*,*D*) The best fitting decision boundaries for rats learning an II task.

Two RB models and one II model were fit to the last five sessions of each rat's training as before. Although it is possible that the rats might switch their categorization strategies throughout training (Hélie et al. 2016), we were mainly interested in which strategy was used upon reaching criterion. The best fitting model was determined according to the AIC values of each model fitting. About half (5/8) of the RB rats were best fit to an RB model and all (8/8) of the II rats were best fit to the II model (Fig. 6). This replicates Experiment 1 in that roughly half of the RB rats used a unidimensional strategy, whereas all of the remaining rats in the RB and II groups used both dimensions to inform their classifications.

## Discussion

The RB and II tasks are identical except for one key manipulation: the RB distributions are perpendicular to one of the two stimulus dimensions, but the II distributions are not. Therefore, the RB tasks, but not the II tasks, contain category-irrelevant information and can be solved on the basis of a single dimension. This distinction creates a potentially important dissociation in how the learner represents the tasks, a dissociation that has been manifested in disparate learning rates (Smith et al. 2012). Indeed, this RB–II framework has become a powerful tool for understanding the dynamics of category learning in multiple species.

In our study, rats learning the RB tasks reached criterion at the same rate as rats learning the II tasks; no differences were observed across training blocks (Figs. 2A, 5B). In Experiment 1, rats generalized their categorization responses to novel exemplars after reaching the learning criterion; no differences in generalization accuracy were observed between the tasks (Fig. 2). Decision boundary analyses also demonstrate that RB rats do not reliably use a RB strategy (Figs. 4, 6). Taken together, these results demonstrate that rat category learning is similar to that of pigeons and dissimilar to that of primates, which learn the RB tasks reliably faster than the II tasks (Smith et al. 2012).

Applying the conceptual framework of Smith et al. (2012) to the current findings suggests several important implications regarding the nature of category learning in rats. First, rats are not true rule users like primates; the ability to test hypotheses and quickly develop category rules was not evident from the current experiments. Second, rats, like pigeons, rely on a single learning system, the procedural system, which incrementally forms S–R connections between-category stimuli and behavioral responses. Finally, because the procedural system does not support executive functions like selective attention, COVIS would predict that both RB and II tasks are learned and represented in the same way, with equal attention being given to both dimensions. From the accuracy data alone, we would conclude that the rats used diffuse attention to learn both tasks, as Smith et al. (2012) suggested for category learning in pigeons.

However, by fitting GCM to the rat's performance, we were better able to estimate how attention was allocated to the two visual dimensions. Importantly, the rats learning the RB tasks were able to find the relevant dimension, and they allocated more attention to that dimension. II rats showed equivalent attention to both dimensions. Therefore, although rats did not exhibit rule use, they did demonstrate selective attention in learning the RB tasks. An alternative interpretation of the GCM analysis is that because the attention weights to the relevant dimension were not 1.0, attention was not truly selective, and rats were therefore using the procedural system to solve the RB task. This alternative interpretation seems unlikely because there is abundant evidence for PFC-mediated selective attention in rats, supporting our view that RB learning in rats can be supported by selective attention to the relevant dimension (Ostlund 2005; Wit 2006; Marquis et al. 2007; Ragozzino 2007; Yoon et al. 2008; Tait et al. 2014, 2018). We therefore propose that rats have the basic attentional mechanism of the declarative system, but this mechanism is not as elaborated as the primate system, which includes rapid rule identification and rule utilization. Thus, the most critical differences between primates and rats in the rate of RB learning are *limitations* in the rat's declarative system, not its *absence*.

The emergence of rule use in categorization is likely to have been a slow evolutionary process. Even though nonhuman primates show a learning advantage for RB over II tasks, their flexibility to use those rules is more limited than that of humans. For instance, when rule complexity increases, like the two-dimensional rules of the XOR problem, monkeys struggle and instead default to strategies consistent with an S–R account (Smith et al. 2004, 2011). This limitation is also apparent in monkeys’ inability to transfer acquired rules in the RB task to new stimuli (Zakrzewski et al. 2018). As a result, Zakrzewski et al. (2018) propose that monkeys lie in the middle ground between humans and pigeons; they have the ability to generate category rules, but they lack flexibility in their use. Thus, a gradient of rule mastery has been proposed, where the emergence of rule use is evident in nonhuman primates, but has continued to develop into the declarative system of humans. Rats may have a declarative system that can use selective attention, but it is more limited than that of nonhuman primates in using category rules.

We predict this qualitative difference between primates and rats stems from elaborations of PFC function. In humans and monkeys, rule generation and utilization have been implicated in the PFC (Asaad et al. 2000; Seger and Miller 2010; Wutz et al. 2018). Neurophysiological studies in monkeys have examined category-selective neurons of the lPFC (Freedman 2001; Freedman et al. 2003; Cromer et al. 2011). These neurons are thought to acquire category rules by organizing S–R associations from the striatum (Antzoulatos and Miller 2011). Once a working rule has been generated, these neurons may exhibit top-down control to regions such as the inferior temporal cortex and the posterior parietal cortex (Freedman et al. 2003; Antzoulatos and Miller 2016). Besides the lPFC, rule use and selective attention in category learning is evident across the PFC, including medial PFC, anterior cingulate cortex, and the ventromedial PFC (Grinband et al. 2006; Seger et al. 2015; Tsutsui et al. 2016; Mack et al. 2017; Bowman and Zeithamova 2018). Assuming that the rat PFC is homologous to the mPFC of primates, we hypothesize that rat selective attention mechanisms mediate RB task learning. We further hypothesize that the development of true rule use in primates results from the addition of the lPFC or some coordinated function of mPFC and lPFC regions to mediate rule identification and utilization.

Lastly, it is generally assumed that humans learn RB tasks faster than II tasks because their developed declarative system is able to quickly find the category rule. Indeed, a specialized declarative system facilitates RB learning; however, prioritizing rule use can be disadvantageous for structures that cannot be solved by rules. A typical human participant will test simple rules to find a quick solution that correctly segregates the categories. When these rules prove unsuccessful, the participant will switch strategies, at which point COVIS predicts control is given to the procedural system. Thus, the longer the participant tests hypotheses, the longer before the participant switches to the optimal strategy; this switching may impair II learning and exaggerate the difference in learning rate between tasks.

Supporting this idea, Filoteo et al. (2010) had participants learn RB and II tasks while completing a concurrent task that disrupted working memory. By increasing the load on the declarative system, a higher proportion of participants used the optimal strategy to learn the II tasks, and performance was facilitated. In the current experiment, the rats learned both tasks at the same rate; however, both tasks were learned very quickly, especially compared to other category stimuli (Wasserman et al. 2012; Brooks et al. 2013; Kim et al. 2018). Although this result would conventionally be interpreted as a deficit or absence of a declarative system, an alternative interpretation is that the rats learned the II tasks faster than expected, as they were able to quickly switch to the optimal II strategy.

To conclude, the COVIS model has had considerable influence on the field of category learning and has inspired a large body of empirical research. Much of the success of COVIS has come from the behavioral dissociations observed when comparing RB and II learning tasks; however, fewer tests have been conducted to validate its neurobiological predictions. The field of category learning would therefore benefit from directly testing these predictions. We recommend rats as an encouraging animal model to investigate these mechanisms because the use of circuit-specific manipulations with optogenetics and chemogenetics may help determine whether there are multiple category learning systems and, if so, then characterize their respective mechanisms.

## Materials and Methods

### Subjects

Long–Evans rats (Experiment 1: *n* = 24 males and 24 females; Experiment 2; *n* = 16 males) were used in the current study. Each rat was individually housed and kept on a 12-h light–dark cycle. All training and testing sessions began at the same time each day (±1 h). Rats had access to water ad libitum. Food was restricted and the weight of each rat was maintained above 85% of its free-feeding weight. The Institutional Animal Care and Use Committee at the University of Iowa approved all procedures.

### Behavioral apparatus

All shaping, training, and testing sessions were conducted within operant chambers (36 × 41 × 36 cm). Each chamber contained a transparent window (13.5 × 10 cm) on the front wall that allowed observation of the rat while inside the chamber. Single 45-mg food pellets were delivered into an aluminum food tray (6.5 × 13 × 4.5 cm) by a rotary pellet dispenser (Med Associates Inc., Georgia, VT, model ENV-203IR). The wall opposite the food tray was outfitted with a LCD flat-screen monitor (Model 1550V, NEC, Melville, NY). An infrared touch screen (15-in, Elo Touch Systems, Fremont, CA) positioned in front of the monitor allowed the rat to interact with images presented on the computer monitor. A relay controller (Model RS-232, National Control Devices, Osceola, MO) permitted communication between the computer and the pellet relay. MATLAB (MathWorks, Natick, MA) was the primary programming software to conduct shaping, training, and testing sessions. A house light positioned above the food tray was always on during sessions. Finally, white noise minimized distraction.

### Handling and shaping

Upon arrival into the animal colony, a 7-d acclimation period with ad libitum access to food and water was given to each rat. Then, food restriction began, and an experimenter handled each rat daily for 1 wk. Body weight was calculated daily as a percentage of the free-feeding weight at the end of the acclimation period. Next, each rat was placed on a laboratory cart (65 × 100 × 83 cm) with twenty 45-mg food pellets scattered on the cart surface. This procedure was repeated daily until the rat consumed all pellets within 15 min, which typically took 5–10 d. Then, each rat underwent a daily shaping procedure to learn to interact with the computer monitor via the touch screen.

### Categorization stimuli

The categorization stimuli were black and white sinusoidal gratings that changed in both spatial frequency and orientation. Spatial frequency ranged from 0.2532 cycles per degree (cpd) to 1.2232 cpd and orientation ranged from 0 to 1.75 radians. These values are within the perceptual limits of rats and were obtained from a pilot experiment to find dimensions of roughly equal salience (Prusky et al. 2002). Linear transformations normalized the dimensions to create a two-dimensional space ranging from 0 to 100. Specifically,

$$\mathrm{Normalized}\;\mathrm{frequency}=\left(\frac{\mathrm{cpd}}{0.0097}\right)-26.10,$$

$$\mathrm{Normalized}\;\mathrm{orientation}=\mathrm{radians}\;\times \;\left(\frac{180}{pi}\right),$$

where cpd is equal to the grating cycles per visual degree.

### Categorization tasks: training

Two categories were created by placing two bivariate normal distributions on the normalized space (Fig. 1A; Category A: X mean = 30, Y mean = 50, X SD = 2.5, Y SD = 20; Category B: X mean = 70, Y mean = 50, X SD = 2.5, Y SD = 20). Each point within the distributions represents a category exemplar with a corresponding spatial frequency value and orientation value, and each distribution constitutes each category. The remaining tasks were generated by rotating these distributions in 45° increments (Fig. 1B–D).

### Trial procedure

Each rat was trained daily on either a RB task or an II task. Each training session contained 80 trials. The rat initiated each trial by touching a star at the center of the screen (Fig. 1E; trial start). Next, a category exemplar (238 pixels × 238 pixels) was randomly generated from the normal distributions and was presented on the screen (cue phase). After three observing touches of the exemplar, the same exemplar was displayed on both the left and right sides of the screen serving as response keys (choice phase). Members of category “A” required a touch to the left response key, whereas members of category “B” required a touch to the right response key. A correct response produced a white box (serving as a secondary reinforcer) and delivered a food reward. An incorrect response initiated a correction trial, where after a 5- to 10-sec timeout, the same trial was repeated from the cue phase. Intertrial intervals ranged from 5 to 10 sec. Training sessions continued until the rat reached a learning criterion (75% accuracy in both categories, on two consecutive sessions). These procedures have been used effectively for category and discrimination learning in rats (Brooks et al. 2013; Kim et al. 2018).

### Category generalization testing

Testing distributions had identical category means as the training distributions, but the SD along the relevant dimension was increased (Fig. 2B; Category A: X mean = 30, Y mean = 50, X SD = 10, Y SD = 20; Category B: X mean = 70, Y mean = 50, X SD = 10, Y SD = 20). For rats in the remaining tasks, these distributions were rotated in 45° increments. By expanding the SD, the testing distributions could be divided into three stimulus types. Some stimuli were within two SDs of the training distributions (Trained). The remaining stimuli went beyond two SDs of the training distributions; these stimuli were either closer to the category boundary or farther away from the category boundary (Proximal and Distal, respectively). Each rat completed five sessions each with 80 exemplars sampled from the testing distributions. The trial sequence was identical to training, except that no correction trials were given on any trials.

### Statistical analysis

Accuracy was defined as the percentage of correct responses during the choice phase (not including correction trials) and was used to evaluate performance each session. Training sessions continued until each rat reached the learning criterion. Using a 2 × 2 between-subjects ANOVA, we examined the effects of sex and task type on the mean number of sessions to reach the learning criterion. In order to equalize the number of samples from each rat and examine accuracy across training, training sessions were vincintized. This procedure systematically arranges sessions into training blocks. For example, vincinitizing 10 sessions into five blocks would simply be averaging two sessions per block. To vincintize sessions that were not evenly divisible by the number of blocks, remainder sessions were added to the center block first and then to outer blocks as necessary (Ratcliff 1979). We vincintized the sessions into five training blocks, as this was the fewest needed sessions for a rat to reach criterion.

We analyzed accuracy during training using linear mixed-effects modeling (R version 3.4.2). The model used included mixed effects for sex, task type, training block, and a quadratic function (across training blocks), as well as random effects for slope, intercept, and the quadratic function. To find the simplest model that fit the data, we used a model simplification strategy (Crawley 2007). Briefly, we started with the full model and then systematically removed random effects one at a time. This process continued until the estimates of the simplified model were significantly different from the larger model before it. Generalization data were fit using the same simplification strategy. The full model included fixed effects for sex, task type, session block, and a quadratic function (across trial blocks), as well as random effects for slope, intercept, and the quadratic function for each rat.

### Decision boundary analysis

The GRT was fit to the generalization data to estimate each rat's decision boundary (Maddox and Ashby 1993). GRT is an extension of signal detection theory and assumes there are two stages of category learning: exemplar encoding and response selection. First, GRT assumes that perceptual events inherently contain noise (resulting from stimulus variability and/or neural noise). Across trials, this variability produces distributions of events. Second, a selection mechanism places a decision boundary among these distributions. These boundaries segregate the space into regions, such that each region is given an exclusive response or label. Multiple selection models exist to construct decision boundaries, and each model assumes a different strategy when partitioning the space. For instance, the unidimensional general linear classifier (GLC) estimates the decision boundary according to a single dimension (i.e., an RB strategy), whereas the bidimensional GLC estimates the decision boundary according to a linear combination of multiple dimensions (i.e., an II strategy).

Three models (i.e., bidimensional GLC, unidimensional GCL using the frequency dimension, and unidimensional GLC using the orientation dimension) were fit to the generalization data. An estimate of goodness of fit was calculated using the AIC statistic [AIC = 2*k* − 2ln(*L*), where *k* is the number of estimated parameters in the model (2 for the one-dimensional GLC, 2 for the two-dimensional GLC) and *L* is the maximum value of the likelihood function (Akaike 1974). The best fitting model for each rat was chosen according to the lowest AIC value.

### Generalized context model

In addition, the exemplar model GCM was fit to each rat's generalization data to gauge how attention was distributed between stimulus dimensions. This model assumes that categorizing some stimulus S involves comparing that stimulus to every exemplar stored in memory (Nosofsky 1986). Then, the probability of assigning S to some category A is the ratio between S's similarity to members of category A and S's similarity to members of all possible categories. To fit GCM to each rat's testing stimuli, we first trained the model using exemplars from the rat's last two training sessions. Then, the MATLAB function fmincon optimized the model's predictions to the rat's responses during testing. GCM has three free parameters: a response bias parameter (*b* > 0), a specificity parameter (*c* > 0), and an attention weight parameter (*w*). Each stimulus dimension is assigned an attention weight 0 < *w* < 1, and all attention weights summed to 1. Additionally, we assumed the dimensions were perceptually separable; therefore, we used the city block distance metric (*r* = 1). We also assumed a Gaussian generalization curve (*P* = 2). Once the model was optimized, the testing stimuli were grouped into 18 bins according to their frequency and orientation values to visualize the model's fit (Fig. 3A; 18 total bins, three per trial type and nine per category). The quality of fit was determined using the coefficient of determination (*R*^2^).

## Supplementary Material

Supplemental Material
