# Supplementary material for: Selective attention in rat visual category learning
Source: Learn Mem. 2019 Mar;26(3):84–92. doi: 10.1101/lm.048942.118 (PMC6380202; doi:10.1101/lm.048942.118)
Supplement: Supplemental Material [file supp_26_3_84__index.html]

Supplemental Material 

# Selective attention in rat visual category learning

## Supplemental Material

- Supplemental1.jpg
- Supplemental2.jpg
- Supplemental\_Legends.docx
