# Supplementary figures and images for: Selective attention in rat visual category learning
Source: Learn Mem. 2019 Mar;26(3):84–92. doi: 10.1101/lm.048942.118 (PMC6380202; doi:10.1101/lm.048942.118)

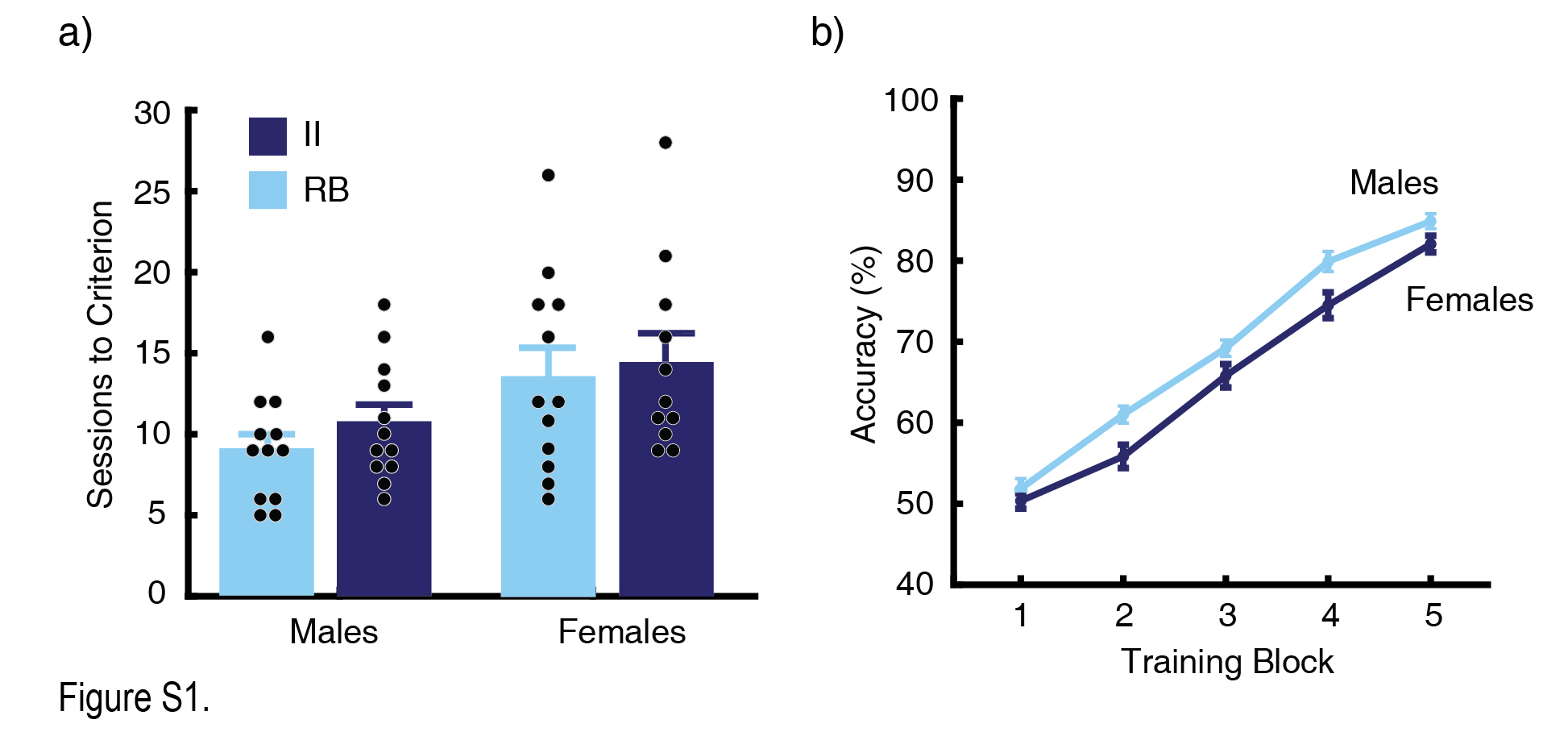

Supplement: Supplemental Material [file supp_26.3.84_Supplemental1.jpg]

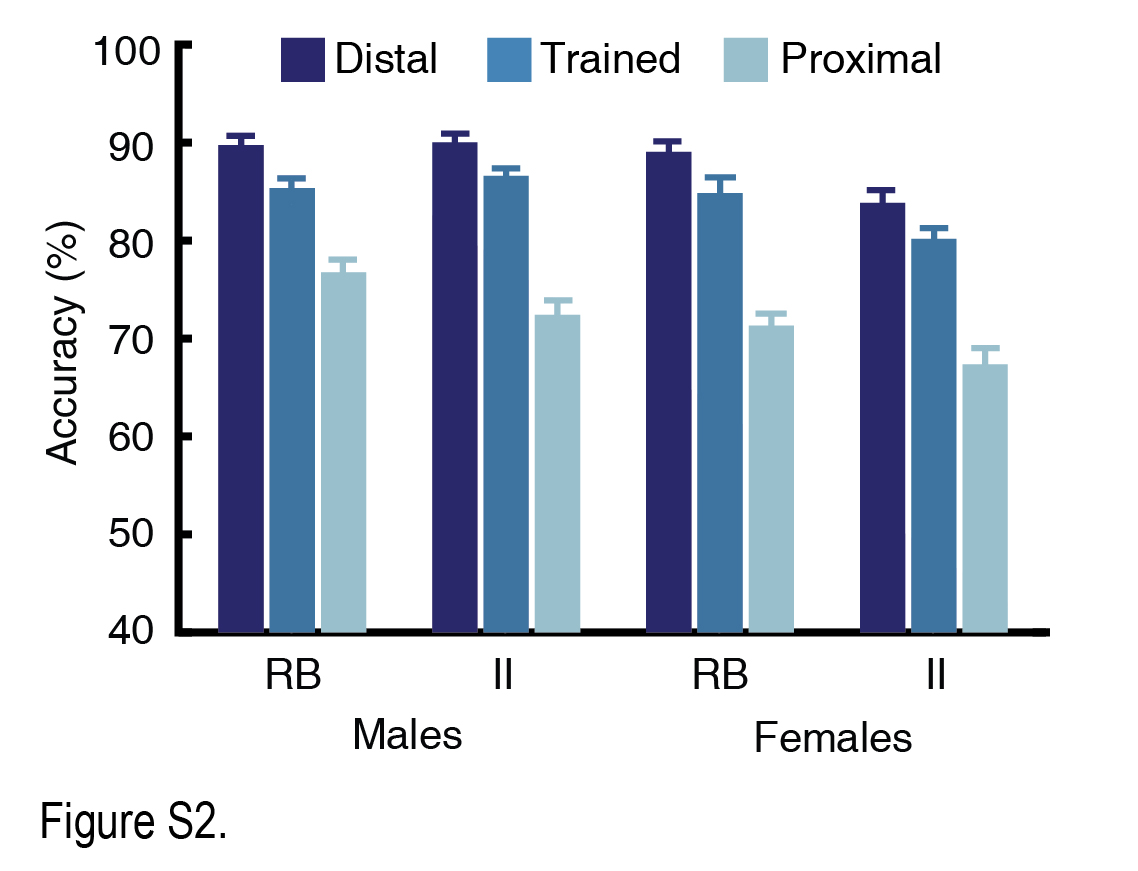

Supplement: Supplemental Material [file supp_26.3.84_Supplemental2.jpg]
