# Supplementary material for: Selective attention in rat visual category learning
Source: Learn Mem. 2019 Mar;26(3):84–92. doi: 10.1101/lm.048942.118 (PMC6380202; doi:10.1101/lm.048942.118)
Supplement: Supplemental Material [file supp_26.3.84_Supplemental_Legends.docx]

**Figure S1.** (a) The mean number of sessions (+/- S.E.M.) for rats to reach the learning criterion. There was no difference in the rate to criterion between rats learning the RB and II tasks; however, there was a significant main effect of sex, such that male rats reached the learning criterion faster than the female rats. (b) Accuracy across training for rats learning RB tasks and II tasks. Training sessions were first vincintized such that each rat’s learning curve was averaged into five training blocks. There was a significant effect of sex, such that across training blocks, males had higher accuracy than females.

**Figure S2.** Category generalization according to sex. For both males and females, accuracy increased for Distal stimuli and decreased for Proximal stimuli compared to Trained stimuli. There was a significant main effect of sex, such that males had higher accuracy than females.
